# Safe Implementation of Treatments in Stroke: a study on intravenous thrombolysis in patients over 80 years of age with acute ischaemic stroke

**DOI:** 10.1136/bmjopen-2024-087454

**Published:** 2025-01-11

**Authors:** Marius Matusevicius, Ana Paiva Nunes, Manju Krishnan, Jose Egido, Letizia Concari, Anand Dixit, Monica Reggiani, Alain Pagès, Thierry Danays, Danilo Toni, Niaz Ahmed

**Affiliations:** 1 Department of Neurology, Karolinska University Hospital, Stockholm, Sweden; 2 Department of Clinical Neuroscience, Karolinska Institutet, Stockholm, Sweden; 3 Stroke Unit, Centro Hospitalar de Lisboa Central EPE, Lisboa, Portugal; 4 Morriston Hospital, Swansea, UK; 5 Hospital Clinico San Carlos, Madrid, Spain; 6 AUSL Parma, Parma, Italy; 7 Newcastle Upon Tyne Hospitals NHS Foundation Trust, Newcastle Upon Tyne, UK; 8 Department of Neurology, Rivoli Hospital, Rivoli, Italy; 9 Boehringer Ingelheim GmbH, Ingelheim am Rhein, Germany; 10 TDC, Aix en Provence, France; 11 Department of Human Neurosciences, University of Rome La Sapienza, Rome, Italy

**Keywords:** STROKE MEDICINE, Stroke, NEUROLOGY

## Abstract

**Abstract:**

**Objectives:**

To investigate the safety and efficacy outcomes of intravenous thrombolysis (IVT) in patients aged >80 years with acute ischaemic stroke (AIS) after IVT was approved in this patient population in several European and non-European countries during 2018–2019.

**Design:**

This is an observational registry study using prospectively collected data from the Safe Implementation of Treatment in Stroke (SITS) registry. Comparisons will be performed between patients treated post-approval (July 2018 to December 2021) period with those treated pre-approval (June 2015 to June 2018) period using propensity score matching (PSM).

**Setting:**

This is a multicentre international study in hospitals treating AIS with IVT.

**Participants:**

Patients aged >80 years who otherwise followed the IVT Summary of Product Characteristics of European countries as part of the mutual recognition procedure.

**Primary and secondary outcomes:**

The main outcomes were symptomatic intracerebral haemorrhage per SITS monitoring study definition, death and functional independency as defined by a modified Rankin Scale score of 0–2 at 90 days.

**Results:**

After PSM, 614 patients remained in each group (mean age 87 years, 39% males). All baseline data were well balanced after PSM. There were no statistically significant differences in outcomes between pre- and post-approval patients for SICH (2.5% vs 2.3%, risk ratio (RR) 1.064, 95% CI 0.345–1.784), death (25.3% vs 28.4%, RR 0.889, 0.699–1.08) and functional independency at 90 days (40.3% vs 37%, RR 1.089, 0.942–1.237).

**Conclusions:**

In this observational study of IVT treatment in patients >80 years of age with AIS before and after formal approval for this treatment, we did not find any difference in outcomes between the pre- and post-approval periods.

**STRENGTHS AND LIMITATIONS OF THIS STUDY**

- Multicentre international study
- Based on intravenous thrombolysis (IVT) summary of product characteristics other than age
- Observational design comparing similar patients over two different periods
- Data unavailable for non-IVT-treated population
- Selection criteria used several steps, decreasing the generalisability.

## Introduction

 Intravenous thrombolysis (IVT) with tissue plasminogen activator (recombinant tissue plasminogen activator, alteplase) within 4.5 hours of acute ischaemic stroke (AIS) onset is an established and approved treatment.^1^,^8^ The effect of IVT is time-dependent, and earlier treatment is associated with better outcomes.^2 3 9^ The use of IVT is increasing but still used in only 10–15% of all patients with ischaemic stroke. Meanwhile, 2–7% of patients treated with IVT suffer from symptomatic intracerebral haemorrhage (SICH) as a complication, leading to death in 1.5–2% and worsening functional outcomes in survivors.^2 3 10^

Until 2018, most European regulatory agencies had not approved IVT with alteplase for use in patients over 80 years of age with AIS, whereas the North American regulatory agency supported IVT in patients over 80 years of age within 3 hours of symptom onset. Despite this, off-label treatment was used in countries that allowed this, raising questions about the underlying safety and equal access to a treatment option.

Pooled analysis of randomised controlled trials (RCTs) of IVT with alteplase for AIS has shown that patients over 80 years of age treated with IVT versus control had equal benefits compared with patients of 18–80 years of age.^3^ The Safe Implementation of Treatment in Stroke-International Stroke Thrombolysis Register (SITS-ISTR) and Virtual International Stroke Trials Archive analysis demonstrated the benefits of IVT in patients over 80 years of age compared with controls,^11^ with similar results when compared with patients under the age of 80 years.^12^

Current European Stroke Organisation guidelines^13^ recommend the use of IVT within 4.5 hours of symptom onset in the elderly, but North American guidelines do not recommend IVT in the 3–4.5-hour time window in patients over 80 years of age.^14^ IVT with alteplase within 4.5 hours of symptom onset is now approved in patients over 80 years of age in a number of European and non-European countries.

As IVT with alteplase in patients over 80 years of age was used off-label in many countries prior to approval, this would merit further study within this growing AIS population to make sure that no unforeseen consequences appear after a wider acceptance of the treatment. Differing on-label requirements for IVT throughout the world could also benefit from this type of analysis.

## Aim

We aimed to compare the safety and outcome parameters in patients over 80 years of age with AIS during a 3-year post-approval period to a 3-year pre-approval period, using criteria for inclusion/exclusion from the regulatory label of IVT using Actilyse (alteplase).

## Methods

This was a retrospective, observational, multinational and multicentre study using the data entered in the SITS-ISTR for analysing patients over 80 years old with AIS treated with Actilyse according to the Summary of Product Characteristics (SmPC) criteria. SITS-ISTR is an ongoing, prospective, multinational and academic-driven registry for centres using thrombolysis for the treatment of patients with AIS. Centres are committed to enter data on consecutively IVT-treated patients with AIS in the registry irrespective of SmPC eligibility criteria.

We planned to include about 1000 patients over 80 years of age with AIS treated with intravenous alteplase within 4.5 hours otherwise according to SmPC in the post-approval period (1 July 2018 to 30 June 2021) and about 1000 in the pre-approval period (June 2015 to June 2018) from approximately 60 European sites. The exact approval dates for each country are listed in online supplemental table 1. In the post-approval period, at least 500 patients were planned to be included from mutual recognition procedure (MRP) countries (Austria, Belgium, Cyprus, Germany, Denmark, Greece, Spain, Finland, France, Ireland, Iceland, Italy, Luxemburg, Malta, Netherlands, Norway, Portugal, Sweden and the United Kingdom). The target of 1000 patients was not reached during the 3-year post-approval period (1 July 2018 to 30 June 2021); therefore, this period was extended to December 2021. The data exchange was stopped when 500 patients registered from MRP countries were achieved. Data were provided to the regulatory authorities as needed. Original ethics approval was obtained from the Stockholm regional ethics committee for this project as part of the SITS-monitoring study (SITS-MOST) II framework (registration number 2022-01157-02), and latest amendment was approved by the Swedish ethics review authority. Ethics approval and patient consent for participation in the SITS-ISTR were obtained in countries where required; the remaining countries approved the register for anonymised audit.

The following data were collected in the SITS-ISTR: baseline and demographic data (age, sex and weight), pre-stroke modified Rankin Scale (mRS) score, blood pressure, laboratory values (plasma glucose and cholesterol), medical history including comorbidities and concomitant medication, results of imaging scans and time logistics. Stroke severity as measured by the National Institute of Health Stroke Scale (NIHSS) and blood pressure data at baseline, 24 hours and 7 days. Imaging scans between 22 and 36 hours or earlier or later if performed. Type of stroke, existence of atrial fibrillation and concomitant medications at 7 days or discharge if occurred earlier than 7 days. At the 3-month follow-up, mRS score, any new events including recurrent stroke (haemorrhagic or ischaemic) and major extracranial haemorrhage.

### Inclusion criteria

Pre- and post-approval periods for inclusion differed for the countries included in the study, based on the following general, country- and centre-specific criteria:

The participants of the study were based on the sites fulfilling the criteria defined and patients treated according to the European Union SmPC of Actilyse during the two periods.

### Country-level criteria

At least 500 patients were registered from European MRP countries. The European MRP countries’ approval was obtained on 1 July 2018, whereby patients included in these countries are defined as pre-approval if the AIS symptoms occurred prior to 1 July 2018 and post-approval if AIS occurred on or after 1 July 2018.

For the non-MRP countries, the first day of the month following the approval date was the start of the post-approval period, with 3 years prior to this date becoming the pre-approval period.

Among MRP countries, one country could contribute a maximum of 30% of individual patient data to the MRP countries. As the total number of patients from MRP countries did not reach 500, we added more patients from Italy which exceeded the 30% limit from one country.

Both the pre- and post-approval periods were planned to be 3 years, but the post-approval period was extended for 6 months to reach the minimum number of patients.

### Centre-level criteria

Centres were considered if at least five patients treated with IVT entered in the SITS-ISTR during the post-approval period, and at least 70% of data were available at the 3-month follow-up in the post-approval period.

### Exclusion criteria at individual patient level

Contraindication(s) to the use of IVT with alteplase per local SmPC for data collected in the SITS-ISTR:

- Low-dose alteplase (eg, 0.6 mg/ kg)
- Treated with endovascular thrombectomy
- IVT with tenecteplase (Metalyse)
- Missing data on SmPC criteria which are collected in the SITS-ISTR (see below).

### List of data collected in the SITS-ISTR and used for exclusion of patients outside the SmPC criteria

- Stroke onset to treatment time >270 min
- Previous stroke within 3 months
- Previous stroke earlier than 3 months+diabetes mellitus
- Anticoagulants, oral
- Heparin for stroke prevention/high dose
- Glucose baseline by mmol <2.7 or >22.2 or by mg/dL <50 or >400
- NIHSS baseline >25
- Minor neurological deficit (baseline NIHSS score ≤3)
- Systolic blood pressure at baseline >185 mmHg
- Diastolic blood pressure at baseline >110 mmHg.

### Outcomes

#### Primary outcomes

- SICH per SITS-MOST definition, defined as parenchymatous haemorrhage (PH) type 2 in the acute ischaemic area or remote PH type 2 at the 22–36-hour post-treatment scan combined with neurological deterioration leading to an increase of 4 points on the NIHSS or death within 24 hours
- Mortality within 90 days
- Functional independency as defined by the mRS score 0–2 at 90 days.

#### Secondary outcomes

- Favourable outcome as defined by the mRS score 0–1 within 90 days
- Secondary safety: SICH per European Australasian Acute Stroke Study 2 definition, defined as any sign of intracerebral haemorrhage including haemorrhagic infarction on any post-treatment imaging scan, combined with neurological deterioration, leading to an increase of 4 points on the NIHSS or death within 7 days
- Time from onset of stroke symptoms to start of IVT treatment/needle time, time from onset of symptoms to door (or as captured in the registry arrival at hospital) and door to needle time.

### Statistical methods

Descriptive statistics (mean and SD for continuous variables, median and IQR for ordinal variables, and percentage and proportion for categorical variables) for baseline and demographic characteristics and outcomes for all included patients are provided separately for pre- and post-approval patients. All time-management variables, baseline NIHSS and pre-stroke mRS score were treated as ordinal variables. To compare differences among baseline, demographic and clinical characteristics between pre- and post-approval patients, standardised mean differences were presented to uniformly compare the imbalance of variables, regardless of the variable nature (numerical and categorical). Propensity score matching (PSM) was the primary statistical approach. Multivariable regression modelling was used for secondary complementary explorative analyses using a binomial distribution with log link as link function to estimate risk ratios (RR) for categorical variables and a Poisson distribution to estimate RR for ordinal variables.

We analysed the outcomes in both unmatched and PSM-matched study populations. The primary analysis used PSM to compare pre- and post-approval patients over 80 years of age. Baseline, demographic, clinical and radiological parameters, as well as treatment logistics and outcomes, were presented. The unmatched study population was composed of all IVT-treated patients over 80 years of age with AIS from both pre- and post-approval periods fulfilling the inclusion and exclusion criteria. The PSM study population was selected using 1:1 greedy nearest neighbour matching using calipers equal to 0.2*SD of the logit of the propensity score. The propensity score was based on the variables listed below. These variables were selected based on established clinical significance and relevant SmPC criteria available in the SITS database: age, sex, pre-stroke mRS score, baseline NIHSS score, systolic blood pressure and glucose level, antiplatelet treatment at stroke onset, history of hypertension, diabetes, atrial fibrillation, hyperlipidaemia, smoking and previous stroke earlier than 3 months, and stroke onset to IVT treatment start time. We included a baseline covariate only if 85% of the patients had a value reported. For baseline NIHSS score and time logistic variables as secondary outcomes, the PSM excluded baseline NIHSS score and onset to IVT, respectively, from the variable list.

The PSM results were presented as RR for binary outcomes and standardised mean differences for ordinal outcomes using pre-approval patients as reference. For our secondary explorative analysis, we used backward stepwise multivariable regression models to find which covariates were most associated with the outcomes. To highlight any effect of approval time, the approval time variable was always included in each final model, whether it was selected in the backward selection model or not. We used a binomial distribution with log as the link function to present RR with their corresponding 95% CIs for categorical outcomes, and a Poisson distribution for RR with their corresponding 95% CIs for ordinal outcomes. We used the same covariates as in the PSM for the secondary analysis.

Missing values were handled by exclusion, meaning a patient missing any SmPC criteria or covariate or outcome for the analyses was excluded.

For the statistical analyses, a p value of <0.05 was considered to be statistically significant. All analyses were performed using R V.4.0.4 (https://cran.r-project.org).

The data that support the findings of this study are available from the corresponding author on reasonable request.

## Results

The study flow chart is shown in online supplemental figure 1. The final study population was 662 patients in the post-approval period and 993 patients in the pre-approval period after inclusion-exclusion criteria were applied. We observed a larger amount of post-approval patients as compared with pre-approval patients who were excluded due to previous stroke within 3 months, data for heparin use for stroke prophylaxis or lack of data within those variables.

Online supplemental table 2 shows the number of patients from each contributing country. For MRP countries, Italy and the United Kingdom contributed the most patients. For non-MRP countries, Estonia and Poland contributed the most.

### Baseline and demographics

The baseline and demographic characteristics of the patients are described in table 1. The post-approval patients had a 2 min longer median onset to door time, a 7 min longer median onset to IVT start time and a 7 min longer door to IVT start time compared with the pre-approval patients. There were slightly more males (5%) and antidiabetic medication (5%) and slightly lower antihypertensive medication (5%) and atrial fibrillation (5%) in the post-approval period as compared with the pre-approval period. Remaining baseline and demographic characteristics, including age and baseline NIHSS, were well balanced between approval periods.

**Table 1:** Baseline and demographic characteristics of patients in the pre- and post-approval periods before matching

Variables	Pre-approvaln=993	Post-approvaln=662	SMD	P value
Age	86.6 (4.2)	86.6 (4.3)	0.014	0.775
Sex, male	34.8 (346/993)	40.0 (265/662)	0.107	0.037
NIHSS, baseline	12 (7–17)	11 (7–17)	–0.073	0.120
Systolic blood pressure, baseline	152 (20.2)	150.2 (19.7)	–0.090	0.072
Diastolic blood pressure, baseline	79.2 (13.3)	78.8 (12)	–0.033	0.511
Glucose, baseline (mmol/L)	7.2 (13.3)	7.3 (12)	0.068	0.177
Onset to door, min	87 (60–126)	89 (62–129)	0.056	0.221
Onset to IVT start, min	144 (105–181)	151 (115–195)	0.159	0.001
Door to IVT start, min	43 (26–71)	50 (30–78.5)	0.117	0.002
mRS, baseline	1 (0–2)	1 (0–2)	0.234	<0.001
Platelet inhibitors, baseline	43.8 (435/993)	40.2 (266/661)	0.072	0.166
Antihypertensive, baseline	78.3 (778/993)	73.6 (487/662)	0.112	0.029
Statin, baseline	32.0 (317/992)	28.9 (189/655)	0.067	0.200
Antidiabetics, baseline	11.4 (113/993)	15.9 (105/661)	0.132	0.010
Hypertension, baseline	78.7 (780/991)	78.4 (518/661)	0.008	0.916
Hyperlipidaemia, baseline	29.2 (287/982)	28.8 (189/656)	0.009	0.900
Diabetes mellitus, baseline	15.1 (150/991)	18.2 (120/661)	0.081	0.119
Smoking, baseline	11.0 (106/966)	11.0 (69/629)	<0.001	1.000
Atrial fibrillation, baseline	26.0 (257/990)	20.8 (137/660)	0.123	0.018
Congestive heart failure	14.8 (146/989)	16.3 (107/656)	0.043	0.434
Previous TIA	6.0 (60/992)	6.5 (43/661)	0.019	0.785
Previous stroke more than 3 months from current stroke	10.0 (99/991)	10.4 (69/661)	0.015	0.832

P -values were calculated by t-test, Mann-Whitney U-test, and Chi-squareχ2 test for continuous, ordinal and categorical variables, respectively. Mean (standard deviationSD), median (inter-quartile range), and percentage (proportion) were presented for continuous, ordinal and categorical variables, respectively. Pre-approval patients were used as a reference for SMD.

IVT, intravenous thrombolysismRS, modified Rankin Scale; NIHSS, National Institute of Health Stroke Scale; SMD, standardised mean differenceTIAtransient ischaemic attack

### Propensity score matching

The baseline characteristics after PSM analysis are shown in table 2. In both groups, 614 patients remained after matching. All baseline and demographic characteristics that were matched were well balanced between the pre- and post-approval periods after PSM. Of the PSM population, 205 and 206 patients were treated within the 3–4.5-hour time window for pre- and post-approval periods, respectively.

**Table 2:** Baseline and demographic characteristics of patients in the pre- and post-approval periods after propensity score matching.

Variables	Pre-approvaln=614	Post-approvaln=614	SMD	P value
Age	86.6 (4.1)	86.6 (4.3)	0.012	0.838
Sex, male	38.6 (237/614)	39.1 (240/614)	0.010	0.907
NIHSS, baseline	10.5 (7–16)	11 (6–17)	0.016	0.916
Systolic blood pressure, baseline	150.4 (20.7)	150.2 (19.6)	–0.009	0.881
Diastolic blood pressure, baseline	78.4 (13.5)	78.7 (12.1)	0.027	0.636
Glucose, baseline (mmol/L)	7.2 (13.5)	7.3 (12.1)	0.045	0.430
Onset to door, min	93 (65–135)	88 (62–128)	–0.085	0.179
Onset to IVT start, min	153 (111.25–195)	151 (114.25–195)	–0.021	0.810
Door to IVT start, min	46 (27–75)	48 (30–79)	0.042	0.275
mRS, baseline	1 (0–2)	1 (0–3)	0.061	0.244
Platelet inhibitors, baseline	41.9 (257/614)	40.9 (251/614)	0.020	0.772
Antihypertensive, baseline	75.4 (463/614)	74.3 (456/614)	0.026	0.693
Statin, baseline	31.5 (193/613)	29.0 (177/611)	0.055	0.370
Antidiabetics, baseline	12.9 (79/614)	15.8 (97/613)	0.084	0.163
Hypertension, baseline	76.5 (470/614)	78.7 (483/614)	0.051	0.411
Hyperlipidaemia, baseline	29.3 (180/614)	28.0 (172/614)	0.029	0.659
Diabetes mellitus, baseline	17.1 (105/614)	18.1 (111/614)	0.026	0.708
Smoking, baseline	10.7 (66/614)	11.2 (69/614)	0.016	0.855
Atrial fibrillation, baseline	18.9 (116/614)	20.2 (124/614)	0.033	0.614
Congestive heart failure	12.9 (79/614)	15.6 (96/614)	0.079	0.192
Previous TIA	5.5 (34/614)	6.7 (41/613)	0.048	0.470
Previous stroke more than 3 months from current stroke	9.6 (59/614)	10.3 (63/614)	0.022	0.775

P- values were calculated by t-test, Mann-Whitney U-test, and Chi-squareχ2 test for continuous, ordinal, and categorical variables, respectively. Mean (standard deviationSD), median (IQRinter-quartile range), and percentage (proportion) were presented for continuous, ordinal, and categorical variables, respectively. Pre-approval patients were used as a reference for Seference for SMD.

IVT, intravenous thrombolysismRS, modified Rankin Scale; NIHSS, National Institute of Health Stroke Scale; SMD, standardised mean differenceTIAtransient ischaemic attack

### Results after PSM

**Figure 1:**
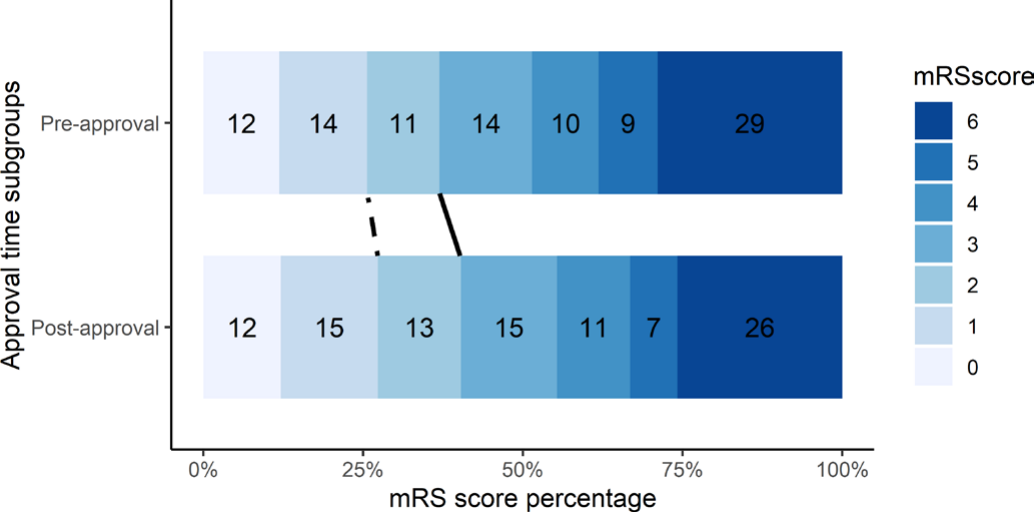
mRS score distribution for the PSM population. Numbers represent percentages. Solid line for limit mRS 0–2 and dotted line for mRS 0–1. mRS, modified Rankin Scale; PSM, propensity score matching.

Table 3 shows the main outcomes after PSM analysis. For our primary outcome, we did not find any statistically significant difference in outcomes between the approval periods. The distribution of mRS score was similar between approval periods within the PSM population (figure 1). Similarly, for our secondary outcomes, there were no statistically significant differences between approval periods.

**Table 3:** Results from the primary analysis of propensity score matching pre- and post-approval patients

Variables	Pre-approvaln=614	Post-approvaln=614	RR or SMD (95% CI)	P value
Primary outcomes				
mRS 0–2 at 3 months	37.0 (205/554)	40.3 (233/578)	1.089 (0.942 to 1.237)	0.280
Death by 3 months	28.4 (161/566)	25.3 (149/589)	0.889 (0.699 to 1.08)	0.254
SICH SITS-MOST	2.3 (14/599)	2.5 (15/603)	1.064 (0.345 to 1.784)	1.000
Secondary outcomes				
mRS 0–1 at 3 months	25.6 (142/554)	27.3 (158/578)	1.066 (0.872 to 1.261)	0.561
SICH ECASS	4.0 (24/594)	5 (30/603)	1.231 (0.707 to 1.756)	0.522
NIHSS, baseline	10.5 (7-16)	11 (6–17)	0.016 (–0.096 to 0.128)	0.916
Onset to door, min	93 (65–135)	88 (62–128)	–0.085 (–0.199 to 0.03)	0.179
Onset to IVT, min	153 (111.25–195)	151 (114.25–195)	–0.021 (–0.133 to 0.091)	0.810
Door to IVT, min	46 (27–75)	48 (30–79)	0.042 (–0.072 to 0.156)	0.275

RR was presented for categorical variables. SMD was presented for ordinal outcomes.

P -values were calculated by Mann-Whitney U-test and Chi-squareχ2 test for ordinal and categorical variables, respectively. Median (IQRinter-quartile range) and percentage (proportion) were presented for ordinal and categorical variables, respectively. Pre-approval patients were used as a reference for RR and SMD.

ECASS, symptomatic intracranial haemorrhage by European Cooperative Acute Stroke Study; IVT, intravenous thrombolysismRS, modified Rankin Scale; NIHSS, National institute of Health Stroke Scale; RR, risk ratio; SITS-MOST, symptomatic intracranial haemorrhage by Safe Implementation of Treatment in Stroke-Monitoring Study; SMD, standardised mean difference

### Results prior to PSM

Online supplemental table 3 shows the main outcomes before PSM analysis. For primary outcome, there was no difference between the approval periods. For secondary outcome, the post-approval period had a longer onset to IVT (median 7 min) and door to IVT start time (median 7 min) compared with the pre-approval period. Other secondary outcomes did not differ between the approval periods.

### Cause of death

Online supplemental table 4 shows the cause of death. Of 429 patients who had died by the 3-month follow-up, cause of death information was available for 416 (97%) patients. The most common cause of death for our study cohort in both pre- and post-approval periods was cerebral infarction, according to International Classification of Diseases 10 diagnosis.

### Explorative analysis

Online supplemental table 5 shows our secondary explorative analyses. The approval period (post- vs pre-approval) was not associated with any SICH definition, favourable outcome or death at 3 months. Patients in the post-approval period had a higher chance of functional independency and lower baseline NIHSS score as compared with patients in the pre-approval period. The post-approval period was associated with longer onset to door, onset to IVT and door to IVT than the pre-approval period.

## Discussion

In this observational study of IVT with alteplase treatment in patients over the age of 80 years with AIS, we did not find any statistically significant differences between pre- and post-regulatory approval of IVT treatment for this patient population regarding our outcomes of death by the 3-month follow-up, functional independence by the 3-month follow-up and SICH per any definition. Similarly, our secondary outcomes did not differ between the compared groups. The results of this study suggest that there was no worsening of outcomes for patients treated off-label before approval, and patients treated after regulatory approval of IVT for patients over 80 years of age with AIS.

Due to the increased use of IVT in AIS, it is important to monitor its effect and evaluate the risk-benefit profile in patient subpopulations. Based on our results, regulatory approval of IVT treatment for patients over the age of 80 years with AIS did not lead to an increase in worse outcomes in these patients as compared with the pre-approval off-label setting. However, the unmatched population showed a longer time of patient’s management in the post-approval cohort compared with the pre-approval cohort which also remained significant in the secondary analysis using multivariable regression models. Despite this, we also observed an increased chance for functional independence in the post-approval cohort as compared with the pre-approval cohort in the secondary analysis, suggesting that there was no apparent worsening and perhaps an improvement of functional outcome during the period after regulatory approval.

IVT treatment is a well-established practice for treating AIS.^6 15^ Yet, the initial IVT trials included very few or no patients above the age of 80 years,^1^ with only inclusion of these patients in the later third international stroke trial (IST-3).^16^ This led to the initial regulatory criteria in Europe that allowed IVT only in patients aged 18–80 years who otherwise fulfilled the SmPC eligibility criteria for IVT.^17^ This has led to questions regarding the safety and efficacy of IVT for patients above the age of 80 years. A previous SITS-ISTR observational study showed that unselected patients over 80 years of age treated with IVT after 3 hours versus within 3 hours had a slightly higher rate of SICH but similar unadjusted functional outcome and poorer adjusted outcome.^18^ One RCT planned to investigate IVT treatment in patients over the age of 80 years but was interrupted early due to the supporting evidence from other RCT that included patients over the age of 80 years.^19^ A meta-analysis of individual patient-level data from seven IVT RCTs compared both IVT treatment between age groups and treatment allocation.^20^ This study also included patients from the SITS in the upper time window monitoring study (SITS-UTMOST), which was an observational study comparing IVT treatment for the extended time window of 3–4.5 hours.^21^ The meta-analysis data found that IVT-treated patients aged >80 years had a higher proportion of mRS 0–1 than non-IVT-treated patients aged >80 years. Our results, both adjusted and unadjusted after PSM, showed higher proportions of patients with mRS 0–1 as compared with the results of the meta-analysis^20^ and in-line with the data from the SITS-UTMOST study.^21^ Importantly, there was no overlap between the patients included in our study and the patients included in the SITS-UTMOST study due to the different periods of inclusion, which allows for comparison between these studies.

Patients older than 80 years have an increased risk of death by the 3-month follow-up than patients under 80 years of age, while maintaining similar safety characteristics regarding SICH.^2022^,^24^ However, this effect of age is not unique to IVT-treated patients and is observed in general in the stroke population, including patients not treated with IVT.^20^ We did not perform an analysis on patients aged 80 years or less in our study. On the other hand, age above 80 years did not show a statistically significant interaction with the effect of IVT treatment in a meta-analysis^20^ suggesting that the effect of age on IVT treatment outcomes may be indirect rather than direct.

One of the prespecified exclusion criteria for this study was ongoing anticoagulant treatment, including direct oral anticoagulants (DOAC). The rationale is that it is an exclusion criterion for IVT treatment due to an assumed heightened risk of SICH. However, recent studies have suggested that this may not be the case. A recent meta-analysis showed that there was no statistically significant difference in haemorrhagic complications in IVT-treated patients with AIS when comparing those who were on DOAC with those who were not on DOAC.^25^ Several recent observational studies have found similar results.^26 27^ While these studies did not specifically investigate patients over the age of 80 years, the median age of their study populations was high and either at or near 80 years of age. Given that the risk of atrial fibrillation and other indications of DOAC increases with age, this subgroup of patients will likely grow given the current demographic shifts towards an ageing population. Therefore, the need to better understand the potential risks of IVT treatment in patients over the age of 80 years will only increase with time.

This study has some limitations. The study design was observational, which limits the possibility of excluding the effect of confounding effects, despite our attempt to minimise this by applying PSM. The study population was defined based on specific inclusion and exclusion criteria, which limits the generalisability of the results. Due to not having data on non-IVT-treated patients, we could not evaluate the potential effect of IVT treatment versus best medical management. Our centre selection criteria may have impacted the results. As we aimed to include centre with high-quality data, we do not know the outcome of patients with poor data quality. The results of this study are derived from an international database with contributions from 15 countries, which increases the generalisability of our results. However, we have applied centre selection based on high data completeness for the 3-month follow-up outcomes. Therefore, centres with poor-quality follow-up are not represented in the analysis. Moreover, patients treated with thrombectomy were not included, and these results may not be applicable to patients treated with a combination of IVT and thrombectomy. Additionally, the post-approval period included patients during the severe acute respiratory syndrome coronavirus 2 pandemic, which could have affected both time parameters and stroke aetiology for patients included during that time.

In conclusion, this observational study did not find any difference in primary or secondary outcomes for IVT-treated patients over the age of 80 years with AIS treated before or after regulatory approval within this subpopulation with AIS. However, we observed some delay in patient management in the post-approval period as compared with the pre-approval period.

## supplementary material

10.1136/bmjopen-2024-087454 online supplemental file 1

## Data Availability

Data are available upon reasonable request.
