# Supplementary material for: Safe Implementation of Treatments in Stroke: a study on intravenous thrombolysis in patients over 80 years of age with acute ischaemic stroke
Source: BMJ Open. 2025 Jan 11;15(1):e087454. doi: 10.1136/bmjopen-2024-087454 (PMC11751842; doi:10.1136/bmjopen-2024-087454)
Supplement: online supplemental file 1 [file bmjopen-15-1-s001.docx]

**Supplementary data elderly**

**Supplementary Figure 1** Study flow chart.

If a patient has missing data on one of the steps (other than steps requiring 2 criteria, where 1 missing and 1 available could be used to classify that patient), that patient was excluded from the entire cohort.

| **Supplementary Table 1.** List of countries and their approval date | | | |
| --- | --- | --- | --- |
| **Country** | **Approval date** | **Start of post-approval period** | **Preapproval period** |
| **EU-MRP** | **2018-Jun** | 2018-07-01 | 2015-06-30 until 2018-06-30 |
| Belgium |  | 2018-07-01 | 2015-06-30 until 2018-06-30 |
| Germany |  | 2018-07-01 | 2015-06-30 until 2018-06-30 |
| UK |  | 2018-07-01 | 2015-06-30 until 2018-06-30 |
| Greece |  | 2018-07-01 | 2015-06-30 until 2018-06-30 |
| Spain |  | 2018-07-01 | 2015-06-30 until 2018-06-30 |
| Italy |  | 2018-07-01 | 2015-06-30 until 2018-06-30 |
| Sweden |  | 2018-07-01 | 2015-06-30 until 2018-06-30 |
| Portugal |  | 2018-07-01 | 2015-06-30 until 2018-06-30 |
| **Non-MRP** |  |  |  |
| Serbia | 2018-Sep | 2018-09-01 | 2015-08-31 until 2018-08-31 |
| Poland | 2018-Nov | 2018-12-01 | 2015-11-30 until 2018-11-30 |
| Bulgaria | 2018-Dec | 2019-01-01 | 2015-12-31 until 2018-12-31 |
| Estonia | 2019-Apr | 2019-05-01 | 2016-04-30 until 2019-04-30 |
| India | 2019-Apr | 2019-05-01 | 2016-04-30 until 2019-04-30 |
| Slovakia | 2020-Jun | 2020-07-01 | 2017-06-30 until 2020-06-30 |
| Iran | 2021-Sep | 2021-10-01 | 2018-09-30 until 2021-09-30 |
| MRP=Mutual recognition process. | | | |

| **Supplementary Table 2.** Final study population per contributing country after applying all inclusion/-exclusion criteria | | | | |
| --- | --- | --- | --- | --- |
|  | **Pre-approval** | | **Post-approval** | |
| **Country** | Number | Percent (%) | Number | Percent (%) |
| **MRP countries** |  |  |  |  |
| Belgium | 39 | 3.9 | 28 | 4.2 |
| Germany | 25 | 2.5 | 20 | 3 |
| Greece | 8 | 0.8 | 29 | 4.4 |
| Italy | 208 | 20.9 | 190 | 28.7 |
| Portugal | 85 | 8.6 | 32 | 4.8 |
| Spain | 67 | 6.7 | 28 | 4.2 |
| Sweden | 65 | 6.5 | 39 | 5.9 |
| United Kingdom | 195 | 19.6 | 135 | 20.4 |
| **Total MRP** | 692 | 69.7 | 501 | 75.7 |
|  |  |  |  |  |
| **Non-MRP countries** |  |  |  |  |
| Bulgaria (2019-01-01) | 11 | 1.1 | 20 | 3 |
| Estonia (2019-05-01) | 162 | 16.3 | 78 | 11.8 |
| India (2019-04-01) | 3 | 0.3 | 0 | 0 |
| Iran (2021-10-01) | 44 | 4.4 | 0 | 0 |
| Poland (2018-12-01) | 70 | 7 | 62 | 9.4 |
| Serbia (2018-09-01) | 0 | 0 | 1 | 0.2 |
| Slovakia (2020-07-01) | 11 | 1.1 | 0 | 0 |
| **Total non-MRP** | 301 | 30.3 | 161 | 24.3 |
|  |  |  |  |  |
| **Total** | 993 |  | 662 |  |
| Percentage for entire pre-approval or post-approval, respectively. Start of post-approval period for non-MRP countries in parentheses.  MRP=Mutual recognition process. | | | | |

| **Supplementary Table 3.** Results from the primary analysis of the unmatched pre-approval and post-approval patients. | | | | |
| --- | --- | --- | --- | --- |
| **Variables** | **Pre-approval**  **N=993** | **Post-approval**  **N=662** | **RR or SMD (95% CI)**  **(Pre-approval as reference)** | **p-value** |
| **Primary outcomes** |  |  |  |  |
| mRS 0-2 at 3-month | 37.1 (332/894) | 39.8 (247/621) | 1.071 (0.942-1.200) | 0.324 |
| Death by 3-month | 28.7 (263/915) | 26.2 (166/633) | 0.912 (0.747-1.078) | 0.303 |
| SICH SITS-MOST | 1.9 (18/965) | 2.3 (15/649) | 1.239 (0.561-1.917) | 0.659 |
|  |  |  |  |  |
| **Secondary outcomes** |  |  |  |  |
| mRS 0-1 at 3-month | 24.9 (223/894) | 26.7 (166/621) | 1.072 (0.899-1.245) | 0.470 |
| SICH ECASS | 4.4 (42/959) | 4.9 (32/649) | 1.126 (0.677-1.575) | 0.692 |
| NIHSS, baseline | 12 (7-17) | 11 (7-17) | –0.073 (–0.171-0.026) | 0.120 |
| Onset to door, minutes | 87 (60-126) | 89 (62-129) | 0.056 (–0.045-0.156) | 0.221 |
| Onset to IVT, minutes | 144 (105-181) | 151 (115-195) | 0.159 (0.060-0.257) | 0.001 |
| Door to IVT, minutes | 43 (26-71) | 50 (30-78.5) | 0.117 (0.017-0.217) | 0.002 |
| RR presented for categorical variables. SMD presented for ordinal outcomes.  P-values calculated by Mann-Whitney U-test, and Chi-square test for ordinal, and categorical variables, respectively. Median (inter-quartile range) and percent (proportion) presented for ordinal, and categorical variables, respectively. Pre-approval patients used as reference for RR and SMD.  The propensity score matching was based on the following variables: Age, sex, pre-stroke mRS score, baseline NIHSS, baseline systolic blood pressure, baseline glucose levels, antiplatelet treatment at stroke onset, history of hypertension, history of diabetes, history of atrial fibrillation, history of hyperlipidaemia, history of smoking, previous stroke earlier than 3 months, and stroke onset to IVT treatment start time.  Abbreviations: RR=Risk ratio, SMD=Standardized mean difference, CI=Confidence interval, mRS=Modified Rankin Scale, SICH SITS-MOST=Symptomatic intracranial hemorrhage by Safe Implementation of Treatment Monitoring Study criteria, SICH ECASS= Symptomatic intracranial hemorrhage by European Cooperative Acute Stroke Study criteria, NIHSS=National institute of Health Stroke Scale, IVT=Intravenous thrombolysis. | | | | |

| **Supplementary Table 4.** Cause of Death, according to ICD-10 Diagnosis for unmatched study cohort. | | | | |
| --- | --- | --- | --- | --- |
| **Cause of Death, based on ICD 10 diagnosis** | **Pre-approval**  **(Total: 258)** | | **Post-approval (Total: 158)** | |
|  | n | % | n | % |
| **Cerebral Infarct** | 92 | 35.7 | 79 | 44.3 |
| **Cerebral Haemorrhage** | 16 | 6.2 | 8 | 5.1 |
| **Cerebral Infarct and Haemorrhage Without Specification** | 16 | 6.2 | 9 | 5.7 |
| **Pneumonia** | 33 | 12.8 | 10 | 6.3 |
| **Pulmonary embolism** | 2 | 0.8 | 1 | 0.6 |
| **Deep Venous Thrombosis** | 6 | 2.3 | 6 | 3.8 |
| **Myocardial Infarction** | 6 | 2.3 | 3 | 1.9 |
| **Other or Unknown Cause of Death** | 87 | 33.7 | 51 | 32.3 |
| Note: Percentages of all deaths within each subgroup presented.  ICD-10= International Classification of Diseases 10^th^ revision. | | | | |

| **Supplementary Table 5.** Results of the secondary analysis with multivariable backwards stepwise regression models. Variables remains in the final model for each outcome are shown in the table. Post vs pre-approval variable was added in the model irrespective if it remained in the final model. | | |
| --- | --- | --- |
| Variables | RR | p-value |
| **Primary outcomes** | | |
| **For mRS 0-2 at 3 months** | | |
| Post vs pre-approval | 1.108 (1.001-1.227) | 0.048 |
| NIHSS at baseline | 0.927 (0.915-0.938) | <0.001 |
| mRS at baseline | 0.757 (0.711-0.806) | <0.001 |
|  |  |  |
| **For death by 3 months** | | |
| Post vs pre-approval | 0.927 (0.775-1.109) | 0.409 |
| Age | 1.012 (0.992-1.032) | 0.246 |
| NIHSS at baseline | 1.079 (1.063-1.096) | <0.001 |
| Pre-stroke mRS | 1.16 (1.09-1.235) | <0.001 |
|  |  |  |
| **For SICH by SITS-MOST** | | |
| Post vs pre-approval | 1.246 (0.613-2.533) | 0.543 |
|  |  |  |
| **Secondary outcomes** | | |
| **For mRS 0-1 at 3 months** | | |
| Post vs pre-approval | 1.135 (0.98-1.314) | 0.091 |
| NIHSS at baseline | 0.91 (0.894-0.927) | <0.001 |
| Pre-stroke mRS | 0.614 (0.556-0.679) | <0.001 |
|  |  |  |
| **For SICH by ECASS** | | |
| Post vs pre-approval | 1.16 (0.718-1.856) | 0.538 |
| NIHSS at baseline | 1.065 (1.026-1.106) | 0.001 |
|  |  |  |
| **For NIHSS at baseline** | | |
| Post vs pre-approval | 0.931 (0.901-0.961) | <0.001 |
| Age | 1.011 (1.007-1.015) | <0.001 |
| Pre-stroke mRS | 1.057 (1.044-1.07) | <0.001 |
| Glucose at baseline | 1.017 (1.009-1.024) | <0.001 |
| Sex | 0.963 (0.932-0.995) | 0.026 |
| Platelet inhibitors at baseline | 0.916 (0.887-0.946) | <0.001 |
| Anti-diabetic medication at baseline | 0.921 (0.874-0.971) | 0.002 |
| History of atrial fibrillation | 1.13 (1.091-1.171) | <0.001 |
|  |  |  |

| **e-Table 5 cont.** | | |
| --- | --- | --- |
| Variables | RR (95% CI) | p-value |
| **Secondary outcomes** | | |
| **For Onset to Door** | | |
| Post vs pre-approval | 1.031 (1.019-1.043) | <0.001 |
| Age | 0.997 (0.996-0.999) | <0.001 |
| NIHSS at baseline | 0.994 (0.993-0.994) | <0.001 |
| Pre-stroke mRS | 1.009 (1.004-1.013) | <0.001 |
| Glucose at baseline | 1.005 (1.002-1.007) | <0.001 |
| Sex | 1.049 (1.037-1.061) | <0.001 |
| Platelet inhibitors at baseline | 0.987 (0.976-0.998) | 0.022 |
| Anti-hypertensive medication at baseline | 1.039 (1.025-1.053) | <0.001 |
| History of atrial fibrillation | 0.983 (0.97-0.996) | 0.013 |
|  |  |  |
| **For Onset to IVT** | | |
| Post vs pre-approval | 1.051 (1.042-1.061) | <0.001 |
| Age | 0.998 (0.997-0.999) | <0.001 |
| NIHSS at baseline | 0.998 (0.997-0.998) | <0.001 |
| Pre-stroke mRS | 0.99 (0.986-0.993) | <0.001 |
| Glucose at baseline | 1.004 (1.002-1.006) | <0.001 |
| Sex | 1.025 (1.016-1.035) | <0.001 |
| Platelet inhibitors at baseline | 1.039 (1.03-1.049) | <0.001 |
| Anti-hypertensive medication at baseline | 1.032 (1.021-1.043) | <0.001 |
| Anti-diabetic medication at baseline | 1.019 (1.004-1.033) | 0.010 |
| History of atrial fibrillation | 0.988 (0.977-0.998) | 0.023 |
|  |  |  |
| **For Door to IVT** | | |
| Post vs pre-approval | 1.088 (1.072-1.104) | <0.001 |
| NIHSS at baseline | 1.005 (1.004-1.006) | <0.001 |
| Pre-stroke mRS | 0.956 (0.951-0.962) | <0.001 |
| Platelet inhibitors at baseline | 1.137 (1.12-1.154) | <0.001 |
| Anti-hypertensive medication at baseline | 1.021 (1.003-1.039) | 0.025 |
| Anti-diabetic medication at baseline | 1.082 (1.059-1.105) | <0.001 |
| Each multivariable backward stepwise regression model started with the same variables as the primary analysis. Variables that are presented had an association with the outcome at p<0.05. In each multivariable model, the binary variable of pre-approval or post-approval was always included into the final model. Pre-approval patients were used as reference in the models.  Abbreviations: RR=Risk ratio, CI=Confidence interval, mRS=Modified Rankin Scale, SICH SITS-MOST=Symptomatic intracranial hemorrhage by Safe Implementation of Treatment Monitoring Study criteria, SICH ECASS= Symptomatic intracranial hemorrhage by European Cooperative Acute Stroke Study criteria, NIHSS=National institute of Health Stroke Scale, IVT=Intravenous thrombolysis. | | |
