# Supplementary material for: Safe Implementation of Treatments in Stroke: a study on intravenous thrombolysis in patients over 80 years of age with acute ischaemic stroke
Source: BMJ Open. 2025 Jan 11;15(1):e087454. doi: 10.1136/bmjopen-2024-087454 (PMC11751842; doi:10.1136/bmjopen-2024-087454)
Supplement: Supplementary file 2 [file bmjopen-2024-087454.reviewer_comments.pdf]

PEER REVIEW HISTORY

BMJ Open publishes all reviews undertaken for accepted manuscripts. Reviewers are asked to complete a checklist review form and are provided with free text boxes to elaborate on their assessment. These free text comments are reproduced below.

ARTICLE DETAILS

Title (Provisional)

Safe Implementation of Treatments in Stroke (SITS) - Intravenous thrombolysis in acute ischaemic stroke patients over 80 years of age study

Authors

Matusevicius, Marius; Paiva Nunes, Ana; Krishnan, Manju; Egido, Jose; Concari, Letizia; Dixit, Anand; Reggiani, Monica; Pagès, Alain; Danays, Thierry; Toni, Danilo; Ahmed, Niaz

---

VERSION 1 - REVIEW

**Reviewer**	**1**
**Name**	**Khalaji, Amirmohammad**
**Affiliation**	**Tehran University of Medical Sciences**
**Date**	**06-Jun-2024**
**COI**	**I have no competing interests.**

The study titled "Safe Implementation of Treatments in Stroke (SITS) - Intravenous thrombolysis in acute ischaemic stroke patients over 80 years of age study" by Matusevicius and colleagues evaluated IVT in patients over 80 years old. The study is well-conducted with fair methodology. I have some comments for improvement:

- Define abbreviations in their first use and make sure that abbreviated forms are being used after the definition.

- Make sure that your figures are color blind friendly.

- Add appropriate subheadings for the results section.

- Mention the strengths of your study before the limitations paragraph.\

- As comorbidities such as AF is higher in older patients (especially >80 years), add a paragraph to the discussion and describe the efficacy of IVT in AIS patients on DOACs. You can use data from recent meta-analyses evaluating IVT in DOAC patients.

- I found some typos and grammatical errors in this version of the manuscript.

**Reviewer**	**2**
**Name**	**Luo, Weiliang**
**Affiliation**	**Huzhou Central Hospital**
**Date**	**12-Aug-2024**
**COI**	**No conflicts of interest**

I think the study is very helpful and the paper is well written. I agree with the authors the study has some limitations, such as some dada collected before the COVID-19 pandemic and some after pandemic which could have affected some parameters.

VERSION 1 - AUTHOR RESPONSE

Reviewer 1
Reviewer's comment/question:
Define abbreviations in their first use and make sure that abbreviated forms are being used after the definition.
Authors' response:
We have updated the manuscript with a definition for each abbreviation the first time it is used, both in the Abstract and in the manuscript separately.

Reviewers' comment/question:
Make sure that your figures are color blind friendly.
Authors' response:
We have used color-blind friendly color palettes for the Figures in the manuscript and Supplementary material.

Reviewer's comment/question:
Add appropriate subheadings for the results section.
Authors' response:
We have added subheadings to the results section for increased clarity.

Reviewer's comment/question:
As comorbidities such as AF is higher in older patients (especially >80 years), add a paragraph to the discussion and describe the efficacy of IVT in AIS patients on DOACs. You can use data from recent meta-analyses evaluating IVT in DOAC patients.
Authors' response:
We agree that this is an important and relevant topic. We have added a paragraph in the Discussion section where we discuss this exclusion criteria for our study, in light of the recent publications regarding DOAC and IVT.

Reviewer's comment/question:

I found some typos and grammatical errors in this version of the manuscript.
Authors’ response:
We have corrected typos and grammatical errors throughout the manuscript.

Reviewer 2
Reviewer’s comment/question:
I think the study is very helpful and the paper is well written. I agree with the authors the study has some limitations, such as some dada collected before the COVID-19 pandemic and some after pandemic which could have affected some parameters.

VERSION 2 - REVIEW

**Reviewer**	**1**
**Name**	**Khalaji, Amirmohammad**
**Affiliation**	**Tehran University of Medical Sciences**
**Date**	**24-Dec-2024**
**COI**	

I have no additional comments.
